# Supplementary material for: Thermodynamic insight into stimuli-responsive behaviour of soft porous crystals
Source: Nat Commun. 2018 Jan 15;9:204. doi: 10.1038/s41467-017-02666-y (PMC5768703; doi:10.1038/s41467-017-02666-y)
Supplement: Supplementary file 1 — Supplementary Information [file 41467_2017_2666_MOESM1_ESM.pdf]

Supplementary Note 1 - MOF names and chemical formula

Supplementary Table 1 given below shows the chemical formula for each MOF mentioned in the manuscript.

Supplementary Table 1: Name and chemical formula of the MOFs mentioned in this work.

MOF Name	***chemical formula***
Al-fum	Al(OH)(fumarate)
CAU-13	Al(OH)(trans-cdc)
MIL-47(V)	V^{IV}(O)(bdc)
MIL-53(Al,Ga,Cr)	(Al,Ga,Cr)(OH)(bdc)
NOTT-300	$Al_2(OH)_2$(bptc)
CPL-2	Cu_2(pzdc)$_2$(bpy)
DMOF-1(Zn)	Zn_2(bdc)$_2$(dabco)
STA-12(Co)	$Co_2(H_2O)_2L$
$Zn(CN)_2$	$Zn(CN)_2$
$Co_3(OH)_2$(btca)$_2$	$Co_3(OH)_2$(btca)$_2$
Co(bdp)	Co(bdp)
Sc_2(bdc)$_3$	Sc_2(bdc)$_3$

Glossary

bdc = 1,4-benzenedicarboxylate	pzdc = pyrazine-2,3-dicarboxylate
cdc = 1,4-cyclohexanedicarboxylate	bpy = 4,4'-bipyridyl
bptc = biphenyl-3,3',5,5'-tetracarboxylate	dabco = 1,4-diazabicyclo[2,2,2]octane
bdp = 1,4-benzenedipyrozolate	btca = benzotriazide-5-carboxylate

L = $O_3PCH_2NC_4H_8NCH_2PO_3$ = N,N'-piperazinebis(methylenephosphonate)

Supplementary Note 2 - Pressure-induced flexibility of UiO-66

The mechanical equation of state as well as the corresponding free energy profile for UiO-66 is given in Supplementary Figure 1 given below, which was taken from Ref. 1.

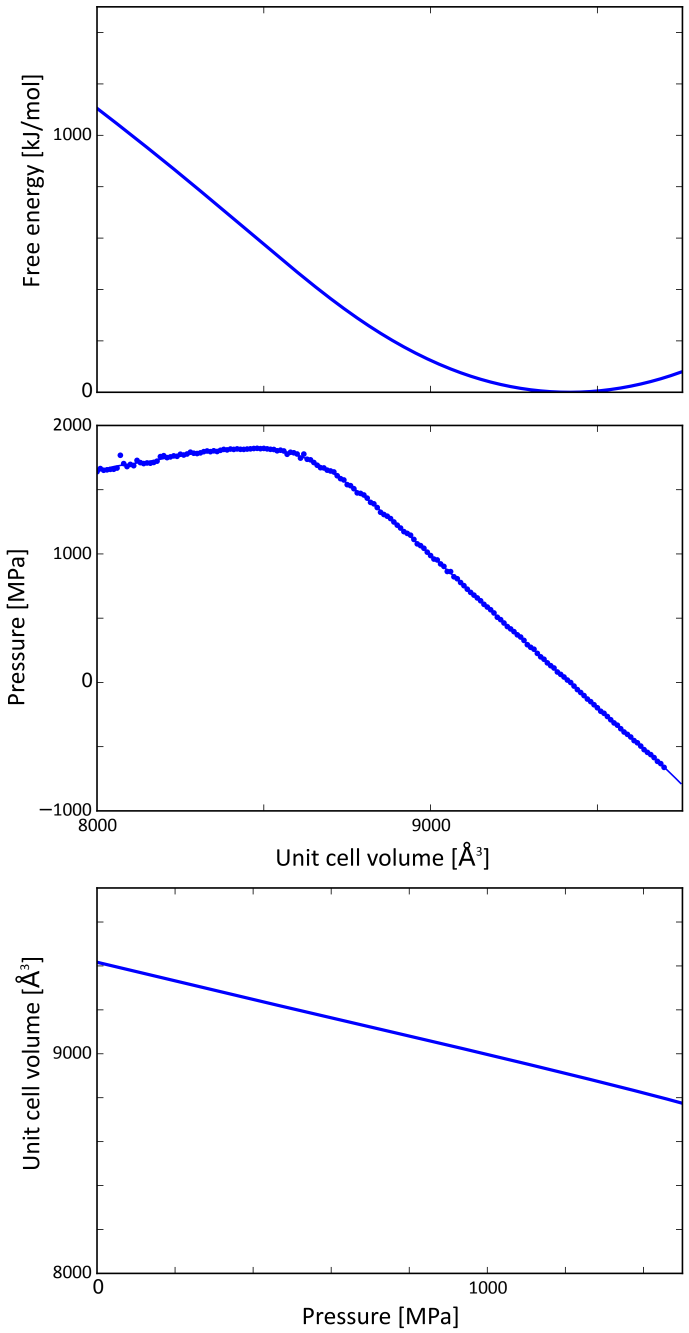

Supplementary Figure 1: Illustration of the mechanical equation of state for UiO-66. Upper pane: free energy profile; middle pane: mechanical equation of state; bottom pane: volume versus pressure. Figure reproduced from Ref. 1.

Supplementary Note 3 - Mechanical energy stored in shock absorbers

As was mentioned in the manuscript, the free energy difference between the contracted pore and the large pore (ΔF) is a lower bound for the work required to induce the large pore to contracted pore transition. Hence, by computing the free energy profile, one can estimate such a lower bounds and rank various shock absorbers according to their capacity to absorb mechanical energy. An approximate method to estimate this mechanical energy stored in the material during one compression/decompression cycle consists of considering transition pressure times the volume change associated with the phase transition ($P_{\text{trans}} \cdot \Delta V$). Supplementary Table 2 applies both methods to MIL-53(Al)-F and DUT-49 to investigate which of the two materials could possibly absorb more mechanical energy.

As can be seen from this table, DUT-49 is able absorb more mechanical energy compared to MIL-53(Al)-F (57.8 J/gr versus 7.9 J/gr). Although the approximate method greatly overestimates the mechanical energy, it does lead to the same qualitative results. Furthermore, we can identify that the larger volume variation for DUT-49 (1.5 Å^3/gr versus 0.4 Å^3/gr) is the cause for the higher energy, since the transition pressure is lower than for MIL-53(Al)-F (60.3 MPa versus 72.4 MPa).

Supplementary Table 2: Comparison of the mechanical energy stored in two shock absorbers, i.e. MIL-53(Al)-F and DUT-49, estimated from the MD profiles (ΔF) as well as using the approximation $P_{\text{trans}} \cdot \delta V$.

	unit	**MIL-53(Al)-F**	**DUT-49**
ΔF	kJ/(mol u.c.)	6.6	1086.6
	J/gr	7.9	57.8
$P_{\text{trans}} \cdot \Delta V$	J/gr	25.6	92.3
mass	gr/(mol u.c.)	840.4	18808.5
ΔV	Å^3/(mol u.c.)	494.2	47820.0
	Å^3/gr	0.4	1.5
P_{trans}	MPa	72.4	60.3

Supplementary Note 4 - Xenon adsorption in MIL-53(Al)

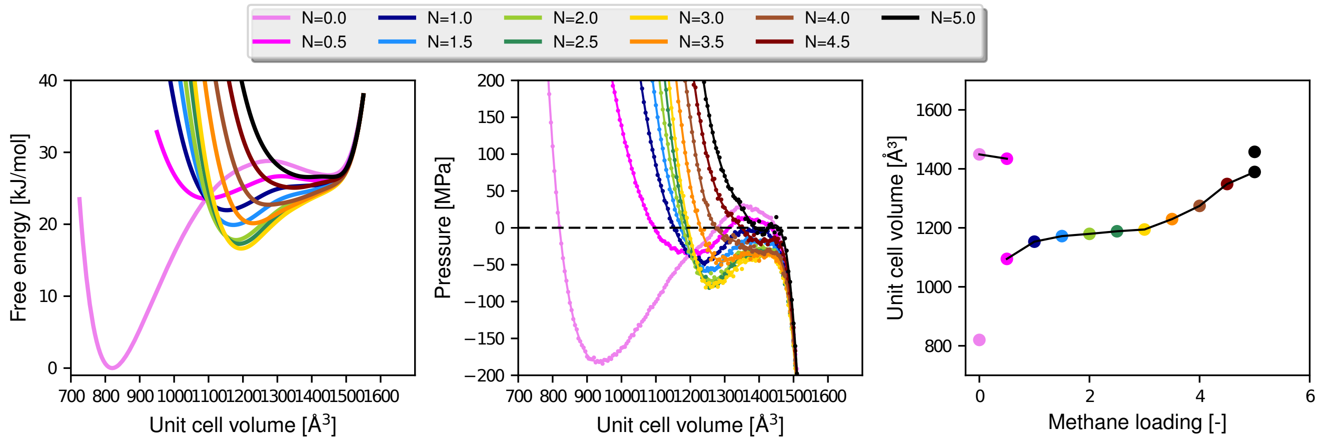

Supplementary Figure 2: Guest induced phase transformations for xenon adsorption in MIL-53(Al). (left) Free energyy (middle) pressure as function of volume for various numbers of xenon atoms adsorbed per unit cell, (right) equilibrium voume as function of the number of xenon particles adsorbed per unit cell.

Guest-induced phase transformations are illustrated for xenon adsorption in MIL-53(Al) in Supplementary Figure 2. Boutin et al. observed breathing transitions in the measured xenon adsorption isotherms for MIL-53(Al) in the temperature range 195-323 K[2]. Under influence of xenon adsorption, the material shrinks to a phase with a volume in between that of the empty contracted pore and large pore phase. We investigated the response of the materials upon guest loading by constructing a series of mechanical equations of state for a fixed temperature but with a varying number of particles (left pane of Supplementary Figure 2). By means of the thermodynamic integration procedure outlined in the manuscript, the free energy profiles are obtained (middle pane of Supplementary Figure 2). Experimentally, one controls the chemical potential, however computationally it is easier to perform molecular dynamics simulations by controlling the number of particles. The obtained profile can be transformed to a equation of state by means of a Legendre transformation of the corresponding Helmholtz free energy to the grand canonical potential[3]. By localizing the stable points at the intersection of the pressure profiles with a fixed pressure of 1 bar, the response curve upon guest loading are constructed (right pane of Supplementary Figure 2). Our model predicts that the material undergoes a phase transition to an intermediate phase with a volume in between the contracted pore and large pore phase, when initially starting from the large pore phase, for a loading of one xenon particle per unit cell. Upon higher loading the volume gradually increases and eventually a transition towards the large pore is observed. This results agree well with the experiments of Boutin et al., who observed breathing transitions in the measured xenon adsorption isotherms for MIL-53(Al) in the temperature range 195-323 K[2] and earlier simulations based on a mean-field model[3].

Supplementary Note 5 - Negative gas adsorption

In this section, we outline how negative gas adsorption can be detected using the thermodynamic model proposed in this manuscript. The starting point of our thermodynamic model is the Helmholtz free energy with state variables temperature T, volume V and number of particles N. However, one can transform one thermodynamic potential to another by means of a Legendre transformation. In an experimental set-up, one typically controls the mechanical pressure on the MOF, the chemical potential of the adsorbent species in the environment and the temperature. In many cases, including in the cases studies discussed below, the mechanical pressure P is equal to the vapor pressure p_{vap} exerted by the gas. The chemical potential and the vapor pressure are coupled by means of the equation of state. To detect the phenomenon of negative gas adsorption one needs to construct the adsorption isotherm $N_T\left(p_{\text{vap}}\right)$ which yields the number of adsorbed particles in terms of the vapor pressure. A negative gas adsorption corresponds to a region where the number of particles decreases when increasing the vapor pressure. This phenomenon was observed for DUT-49 by the group of Kaskel, as shown in Supplementary Figure 3[4].

The thermodynamic model as presented in our paper, yields the volume response with respect to an external

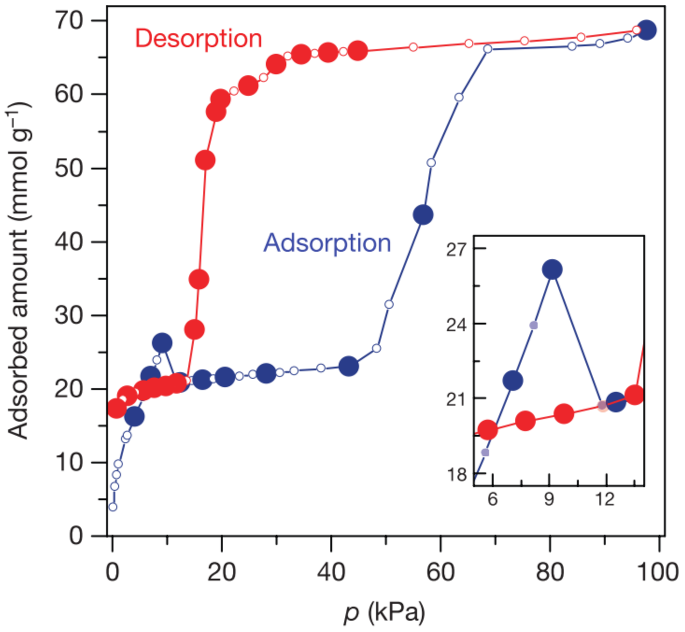

Supplementary Figure 3: adsorption isotherm for methane in DUT-49 at 111 K. Reprinted with permission from Ref. 4. Copyright (2017) Nature Publishing Group.

stimulus (third row in Figure 4 of the manuscript) and yields direct information on the framework flexibility in terms of an external stimulus but does not allow to directly detect the phenomenon of negative gas adsorption. To obtain such information one needs to transform the $F_T(V;N)$ curves towards the thermodynamic potential in the osmotic ensemble in terms of the control variables (μ, P, T) by means of a Legendre transformation:

$$X_T\left(\mu, P\right) = \min_{(N,V)} \left\{F_T\left(V;N\right) + PV\mu N\right\} \tag{1}$$

Such procedure was performed by Vanduyfhuys et al.[3] In the latter paper, the minimization was performed in two steps: first a minimization is performed in terms of the number of particles for fixed values of μ and P:

$$\frac{\partial \tilde{X}_T}{\partial N} = 0 \tag{2}$$

imposing chemical equilibrium, where $\tilde{X}_T\left(N, V; \mu, P\right) = F_T\left(V;N\right) + PV - \mu N$ is an intermediate potential in which N and V are treated as independent variables and μ and P are fixed parameters. As a result the thermodynamic potential is obtained $\tilde{X}_T\left(V;\mu, P\right)$, in which the chemical potential indeed acts as a trigger, but also the amount of adsorbed guest particles $N_T\left(V;\mu, P\right)$. As was mentioned before, the mechanical pressure P equals the vapor pressure p_{vap}. Therefore, from now on, we will drop either μ or P from the argument list of $\tilde{X}_T$

and N_T and assume μ to be a function of p_{vap} through the equation of state. Furthermore, the pressure term PV in $\tilde{X}_T$ can be neglected for vapor pressures in the order of 1 bar. In the following, we ignore this term and as a result, the intermediate potential $\tilde{X}_T(V;\mu,P)$ actually becomes the grand canonical potential $F_T(V;\mu)$ in the (μ,V,T) ensemble.

We illustrate the procedure for xenon adsorbed in MIL-53(Al), for which the $F_T(V;N)$ profiles were computed in Supplementary Note 4 of this supporting information. Supplementary Figure 4(a) shows the $\tilde{X}_T(V;p_{\text{vap}})$, in terms of the volume whereas Supplementary Figure 4(b) shows the number of adsorbed guest molecules in terms of the volume for various values of the vapor pressure. In principle one needs to perform the second minimization step in terms of the volume on this thermodynamic potential. By applying a same procedure as explained in Figure 5 of the main paper on $\tilde{X}_T(V;\mu)$, but with a mechanical pressure given by the vapor pressure $P_0 = p_{\text{vap}}(\mu)$ instead of the atmospheric pressure $P_0 = 1$ bar, one obtains the volume response with respect to the chemical potential (or vapor pressure), which is visualized for the case of xenon adsorbed in MIL-53(Al) in Supplementary Figure 5(a). We now rationalize how the profiles of $\tilde{X}_T(V;p_{\text{vap}})$ and $N_T(V;p_{\text{vap}})$

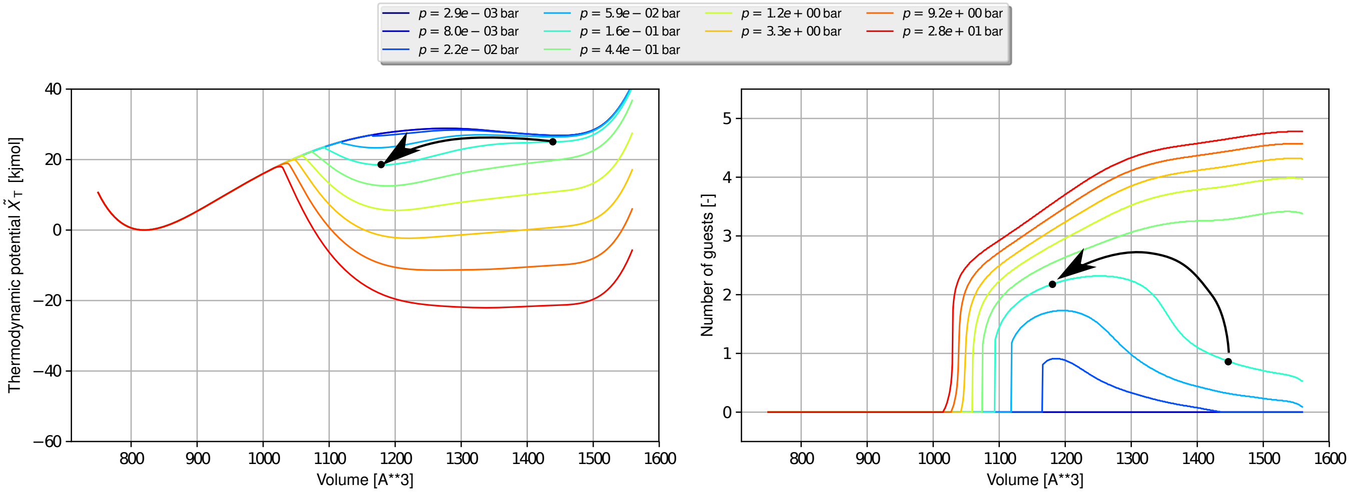

Supplementary Figure 4: Results for applying the first Legendre transform of number of particles N to chemical potential $\mu = \mu(p_{\text{vap}})$ for xenon in MIL-53(Al). (a) thermodynamic potential $\tilde{X}_T(V;p_{\text{vap}})$ and (b) number of particles $N_T(V;p_{\text{vap}})$ for various values of the vapor pressure p_{vap}

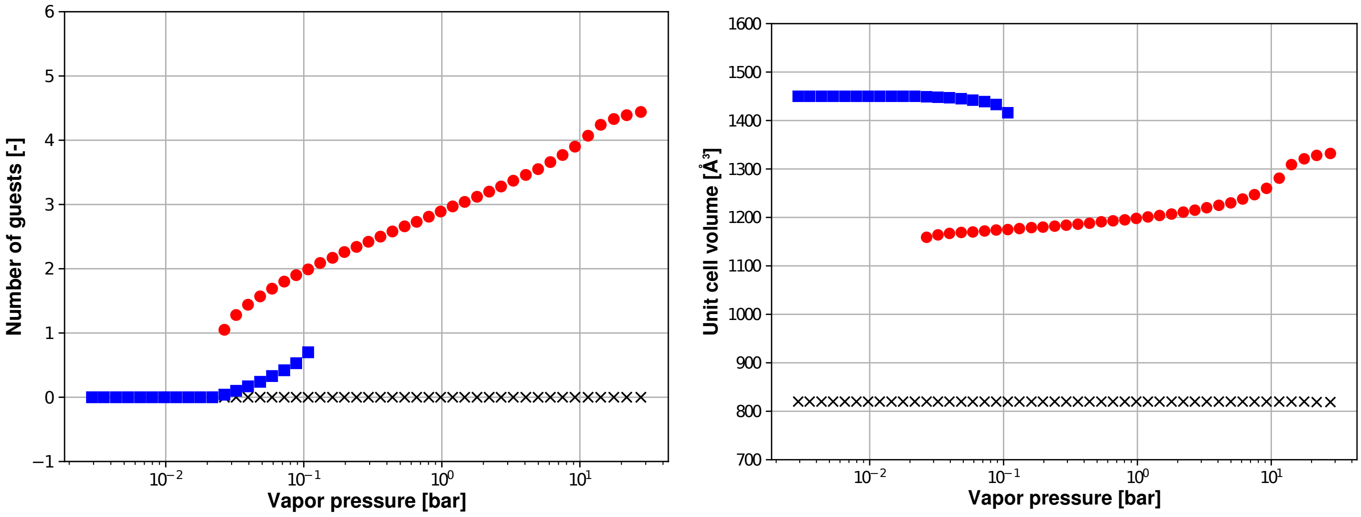

Supplementary Figure 5: Results for applying the second Legendre transform of volume V to mechanical pressure $P = p_{\text{vap}}$ for xenon in MIL-53(Al). (a) volume response to vapor pressure (b) adsorption isotherm. Blue squares represent large pore, red circles represent contracted pore and black crosses represent empty closed pore.

look in case of positive and negative gas adsorption. Suppose we start for the xenon adsorbed in MIL-53(Al)

initially in the large pore phase. This corresponds to most-experimental sample preparations so far, since the material is outgassed at high temperature. When increasing the vapor pressure, a new minimum appears at an intermediate phase. The transition from the large to the intermediate phase only occurs when the large pore minimum in the osmotic potential disappears. Further increasing the vapor pressure finally yields back the large pore phase. In this case positive gas adsorption in observed, as can be detected by inspecting the $N_T\left(V;p_{\mathrm{vap}}\right)$ profiles. As the vapor pressure increases, the structure transforms at a vapor pressure of 0.1 bar (see arrow for the profile at 0.16 bar in Supplementary Figure 4). At this transition, the number of particles increases in case of the xenon in MIL-53(Al). To truly compare with experiment one can perform the second minimization step of the Legendre transformation and construct the adsorption isotherm. The results of this second minimization step for xenon in MIL-53(Al) are shown in Supplementary Figure 5(b).

To test the hypothesis further we started from the free energy profiles published by Evans et al.[5] (see Sup-

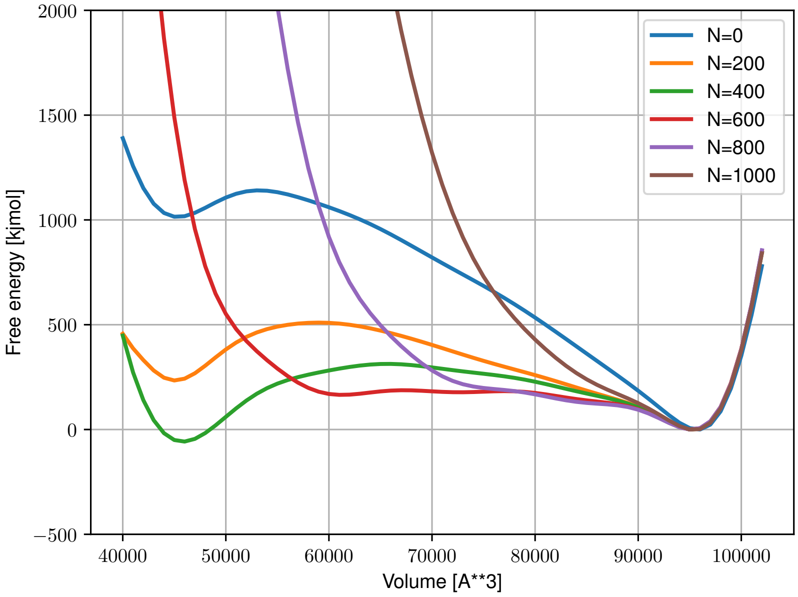

Supplementary Figure 6: $F_T\left(V;N\right)$ profiles computed by Evans et al.[5]

plementary Figure 6). Following the procedure with the Legendre transform sketched above, Supplementary Figure 7 shows the results of applying the first Legendre transform to these profiles, i.e. the thermodynamic potential $\tilde{X}_T\left(V;p_{\mathrm{vap}}\right)$ as well as the number of particles $N_T\left(V;p_{\mathrm{vap}}\right)$ for various values of the vapor pressure p_{vap} of methane. From these results, one would also conclude that no structural transition is observed when collective behavior is assumed and the system always remains in the large pore, because this large pore minimum never disappears. However, it could be that the transition is not observed due to missing data in the original profiles. Looking at the $F_T\left(V;N\right)$ profiles in Supplementary Figure 6, extra $F_T\left(V;N\right)$ curves for a number of methane molecules between 400 and 600 could prove crucial, because, as Evans et al. mention, that is exactly the range in which the barrier between large and narrow pore decreases and hence where transitions could happen. From Supplementary Figure 7(a), we could hypothesize, however, that if a structural transition would occur, it will be at a vapor pressure of around 7.4 bar (see arrow in Supplementary Figure 7), because that corresponds with the thermodynamic potential with the lowest barrier. Furthermore, if we now consider Supplementary Figure 7(b), we observe that at 7.4 bar, the large pore already contains more methane than the narrow pore, so if the transition from large pore to narrow pore indeed occurs, the amount of adsorbed methane would decrease. In other words, negative gas adsorption would occur. To further illustrate this, Supplementary Figure 8(a) also shows the response of the volume to the vapor pressure of methane and Supplementary Figure 8(b) shows the adsorption isotherm of methane in DUT-49. As we can see in the adsorption isotherm, a strong increase in the amount of adsorbed methane for the large pore (blue squares) occurs at around 6 bar, and as a result the amount of adsorbed methane in the large pore (blue squares) becomes larger than in the narrow pore (red circles). Hence, if the transition occurs at pressures larger than 6 bar, negative gas adsorption would indeed occur.

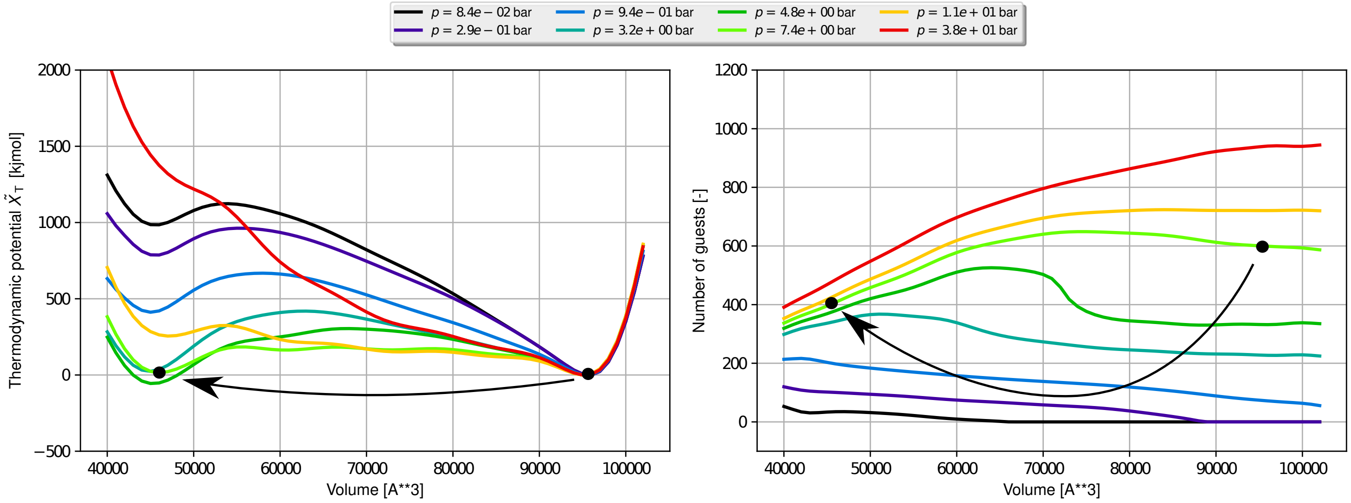

Supplementary Figure 7: Results for applying the first Legendre transform of number of particles N to chemical potential $\mu = \mu(p_{\text{vap}})$ for methane in DUT-49, for which the $F_T(V;N)$ profiles were taken from Ref.[5]. (a) thermodynamic potential $\tilde{X}_T(V;p_{\text{vap}})$ and (b) number of particles $N_T(V;p_{\text{vap}})$ for various values of the vapor pressure p_{vap}

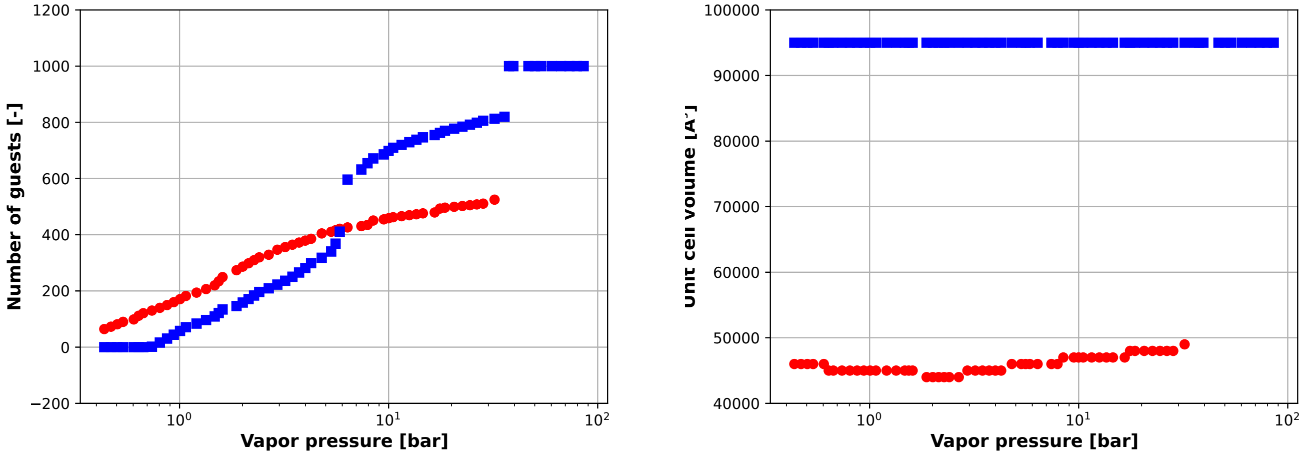

Supplementary Figure 8: Results for applying the second Legendre transform of volume V to mechanical pressure $P = p_{\text{vap}}$ for methane in DUT-49, for which the $F_T(V;N)$ profiles were taken from Ref.[5]. (a) volume response to vapor pressure (b) adsorption isotherm. Blue squares represent large pore, red circles represent contracted pore.

Supplementary Note 6 - Force field derivation

The force fields for MIL-53(Al), MIL-53(Ga), Al-fumarate, MIL-53(Al)-F and Co(bdp) were newly derived from periodic *ab initio* data using QuickFF[6], taking an *a priori* contribution into account for the electrostatic interactions, while the van der Waals contribution from the MM3[7,8] force field was added *a posteriori*. For DMOF-1 several force fields were compared (see Supplementary Note 7), including the force field of Grosch and Paesani[9], UFF4MOF[10] as well as a QuickFF-derived force field according to the procedure outlined below. The UFF4MOF force field for DMOF-1 was derived using the tool from Boyd et al.[11]. For DUT-49 the force field of Evans et al.[5] was used.

Ab initio input

The required *ab initio* data consists of the geometry and Hessian in equilibrium and has been computed via periodic DFT calculations with VASP[12]. The exchange-correlation energy was calculated with the PBE functional[13,14] and D3 dispersion interactions were taken into account together with the Becke-Johnson damping scheme (DFT-D3(BJ))[15,16]. A Gaussian smearing ($\sigma = 0.05$ eV) scheme was applied. First, an energy profile as a function of volume was constructed around a stable phase. The equilibrium volume was obtained by fitting the energy versus volume to the Rose-Vinet equation-of-state (EOS)[17]. This approach has been shown to deliver good results for flexible materials[18]. Finally, after relaxing the structure at the equilibrium volume, the Hessian was calculated using a finite difference approach. Two displacements of 0.01 Å for every cartesian degree of freedom were used.

For the MIL-53-like materials, a plane wave energy cutoff of 500 eV is employed. A 2x6x2 k-point grid is used, with 6 k-points along the metal-oxide chain. The electronic (ionic) convergence criterium is 10^{-8} (10^{-7}) eV. We used the conventional MIL-53 unit cell containing 76 atoms for MIL-53(Al), MIL-53(Ga), 72 atoms for MIL-53(Al)-F and 52 atoms for Al-fumarate.

For DMOF-1, we used a plane wave energy cutoff of 600 eV. A 2x2x2 k-point grid was used for a 1x2x1 supercell containing 216 atoms. The unit cell was doubled along the inorganic chain. The electronic (ionic) convergence criterium is 10^{-8} (10^{-6}) eV.

For Co(bdp), we used a plane wave energy cutoff of 600 eV and a k-point grid of 1x1x3 with 3 k-points along the cobalt chain. Our computational unit cell contains 100 atoms. We took spin polarization into account with a total magnetic moment of 12 per unit cell, as every cobald atom has 3 unpaired electrons. Here, we assume a ferromagnetic interaction. The electronic (ionic) convergence criterium is 10^{-8} (10^{-6}) eV.

Electrostatic contribution

Electrostatic interactions between all atom pairs are included and they were modeled by means of coulomb interactions between fixed gaussian smeared charges. These charges were derived using the Minimal Basis Iterative Stockholder partitioning scheme[19] from the electron density obtained via a single-point GPAW[20,21,22] calculation on the VASP equilibrium structure. The gaussian radii were computed according to the scheme of Chen and Martínez[23]

Covalent contribution

The covalent energy expression of the force field consists of the original QuickFF expression (i.e. harmonic bonds, harmonic bends, single cosine dihedrals and harmonic out-of-plane), augmented by stretch-stretch and

stretch-angle cross terms:

$$V = \sum_m \frac{K^r_m}{2} \left(r_m - r_{0,m}\right)^2 + \sum_n \frac{K^\theta_n}{2} \left(\theta_n - \theta_{0,n}\right)^2 \tag{3}$$

$$+ \sum_p \frac{K^\psi_p}{2} \left[1 - \cos\left(m_p\psi_p - m_p\psi_{0,p}\right)\right] + \sum_q \frac{K^d_q}{2} \left(d_q - d_{0,q}\right)^2 \tag{4}$$

$$+ \sum_{ijk} K^{rr}_{ijk} \left(r_{ij} - r_{0,ij}\right)\left(r_{jk} - r_{0,jk}\right) + \sum_{ijk} \left(\theta_{ijk} - \theta_{0,ijk}\right) \left[K^{r\theta 1}_{ijk} \left(r_{ij} - r_{0,ij}\right) + K^{r\theta 2}_{ijk} \left(r_{jk} - r_{0,jk}\right)\right] \tag{5}$$

Furthermore, for bending patterns for which a rest value of both 90° as well as 180° is possible, a cosine term V^{sq} is used instead of a harmonic term, and in that case no stretch-angle cross terms are included. To avoid the singularity in the derivative in case of an angle of 180° for bending patterns with a rest value of 180°, a term harmonic in the cosine of the angle is used instead of harmonic in the angle itself, and again no stretch-angle cross terms are included.

$$V^{\theta,sq}_n = \frac{K^{\theta,sq}_n}{2} \left[1 - \cos\left(4\theta_n\right)\right] \tag{6}$$

The force constants of the cross terms were estimated simultaneously with the refinement of the diagonal force constants by minimizing the least-squares cost function that measures the error between the *ab initio* Hessian and the force field Hessian in cartesian coordinates. After obtaining a complete covalent force field, the perturbation trajectory of each harmonic internal coordinate is revisited, but now the contribution of all other internal coordinates is taken into account and is subtracted from the *ab initio* energy, and the rest value and force constant are extracted by fitting a parabola to the updated trajectory. Finally, the least-square fitting was performed one last time to refine all force constants. All force field parameters are provided in the Yaff parameter file format in the online supplementary information.

In the case of Co(bdp) and DMOF-1 some negative frequencies remained present in the *ab initio* Hessian, even after repeatedly perturbing the system in the direction of these negative frequencies. Therefore, we explicitely projected these negative frequencies out to the *ab initio* Hessian, prior to fitting a force field to it using QuickFF. Furthermore, we investigated the impact of these negative frequencies on the force field parameters by also fitting a force field to the raw Hessian prior to projecting the negative frequecies out of it, and no substantial differences were found in the force field parameters.

Supplementary Note 7 - Influence of the force field

In this section, we investigate the sensitivity of the free energy profiles of DMOF-1 and the conclusions drawn from it with respect to the applied force field. For this material, we used the force field of Grosch et al.[9] in the manuscript. In this force field, the covalent terms that describe interactions within the BDC and DABCO ligands are taken from the the Generalized Amber Force Field (GAFF)[24]. Covalent interactions involving the metal unit are parametrized using DFT calculations using the M06-2X functional on framework fragments[25]. Electrostatic interactions are described using point charges, which are obtained by fitting to the ab initio electrostatic potential (from the DFT framework-fragment calculations) using the CHELPG method[26]. The van der Waals interactions are modeled using a Lennard-Jones potential using the corresponding GAFF parameters. We now also generated a QuickFF force field for the DMOF-1 framework, starting from an ab initio determined Hessian using periodic Density Functional Theory calculations (see Supplementary Note 6). Furthermore we also applied a general purpose force field namely UFF4MOF[10]. UFF4MOF was developed as an extension of UFF towards MOFs, with new parameters specifically valid for MOF materials. UFF4MOF has been shown very accurate in reproducing unit cell dimensions, however it needs to be tested in how far the parameter set is accurate enough to simulate physical phenomena that are more sensitive to the specific shape of the potential energy surface and temperature corrections such as breathing. A recent assessment of various force fields to predict bulk materials properties like the bulk modulus and linear thermal expansion for a series of MOFs was performed by Boyd et al.[11] Their study only included rigid materials like IRMOF-1, IRMOF-10, HKUST-1 and UiO-66.

The results of the thermodynamic model are given in Supplementary Figure 9 for the three tested force fields. All force fields predict that the volume decreases as the benzene loading increases. The results of the QuickFF and the Grosch force field are the closest together. The UFF4MOF forcefield predicts substantially different results than the other two force fields. UFF4MOF predicts a second branch in the $V(N)$ profile, which is the most stable as soon as two or more benzene molecules are present per unit cell. This branch shows volumes around 1900 3 which is not in agreement with experiment. The results of QuickFF and the Grosch forcefield agree within acceptable limits. The QuickFF predicts in general larger volumes than the other force fields as well as experiment. Note however that for zero loading it is close to the PBE volume to which it was fitted. Grosch et al. shows a more pronounced decrease in volume after a loading of 2 benzene molecules per unit cell. This is accompanied by a transition from a square to a rectangular cell. For QuickFF, the cell remains (more or less) square.

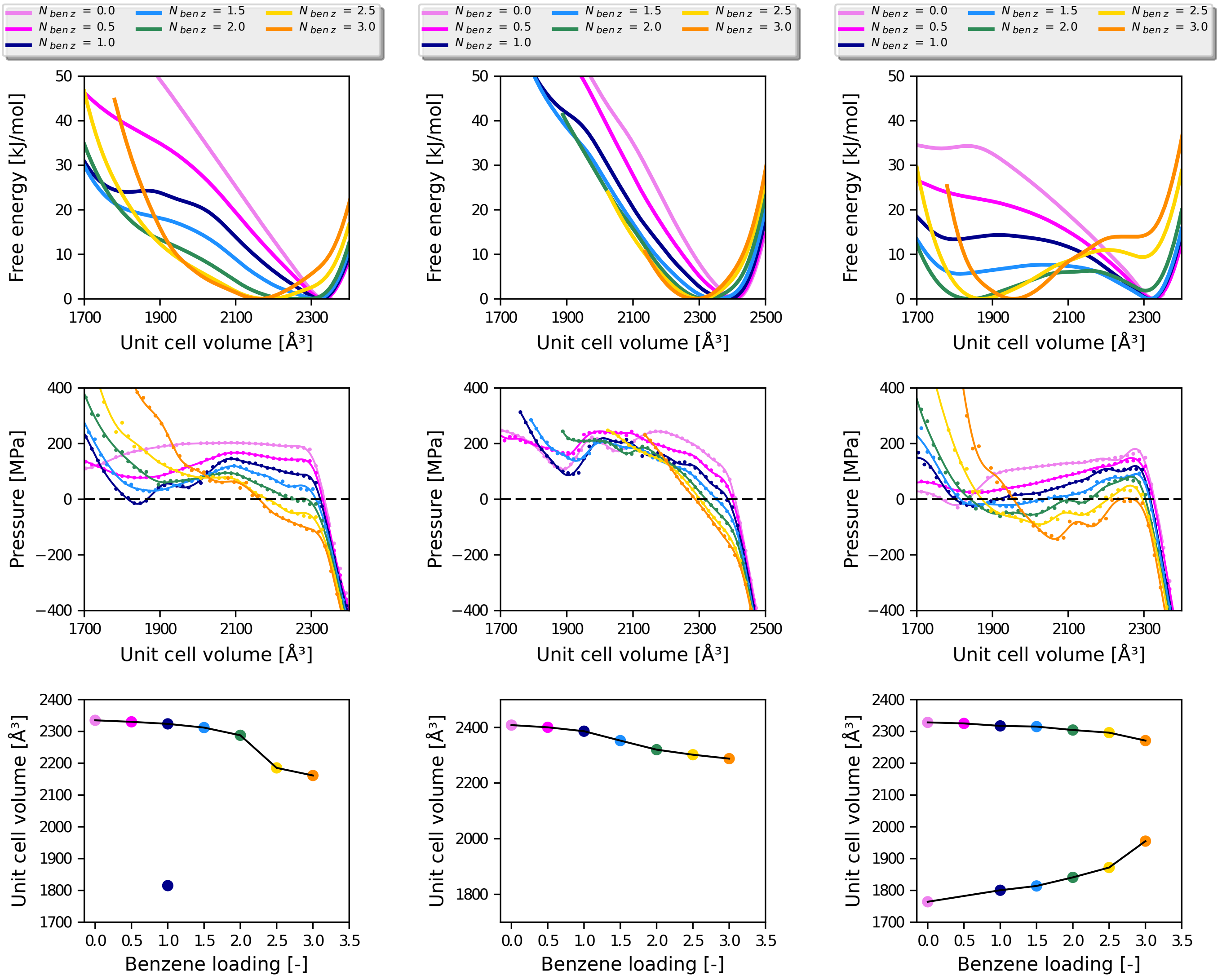

Supplementary Figure 9: Illustration of the mechanical equations of state for benzene adsorbed in DMOF-1 generated with three different force fields. (left) with the force field of Grosch et al.[9], (middle) with an in-house developed force field generated by the QuickFF routine[6] and (right) with a general purpose force field UFF4MOF[10]. The various profiles plotted are: (upper row) free energy profile, (middle row) mechanical equations of state and (Bottom row) volume versus number of adsorbed benzene molecules.

Supplementary Note 8 - Computational details of MD simulations

The molecular dynamics (MD) simulations reported in the main text were carried out in the $(N, V, \boldsymbol{\sigma}_a = \mathbf{0}, T)$ ensemble to generate the different pressure-versus-volume curves, and in the $(N, P, \boldsymbol{\sigma}_a = \mathbf{0}, T)$ ensemble to generate the initial configurations used in the $(N, V, \boldsymbol{\sigma}_a = \mathbf{0}, T)$ simulations (see Ref. 27 for the terminology). Except for DUT-49(Cu), all MD simulations were carried out using Yaff, a freely available in-house developed software package[28]. For DUT-49(Cu), Yaff was interfaced with pydlpoly, which is a DL_POLY Classic[29] wrapper developed by Schmid and coworkers[30]. To construct the data of Figure 4(a,b) of the manuscript, $2 \times 3 \times 2$ super cells were used, except for MIL-53(Al)-F, for which a $2 \times 4 \times 2$ super cell was considered (the conventional unit cell of MIL-53(Al) contains 76 atoms) and DUT-49(Cu), for which a single unit cell containing 1728 atoms is used. In Figure 4(c,d) of the manuscript, a $2 \times 4 \times 2$ super cell was considered for DMOF-1, while a single unit cell is used for Co(bdp). For the simulations of xenon in MIL-53(Al) presented in Supplementary Note 4 of, a $1 \times 2 \times 1$ super cell was used.

To ensure energy conservation during the velocity Verlet update scheme, a time step of 0.5 fs was used during the MD simulations for all materials except for DMOF-1 and Co(bdp), where a time step of 1.0 fs and 0.75 fs was used respectively. The electrostatic interactions were efficiently calculated using an Ewald summation with a real-space cutoff of 15 Å, a splitting parameter α of 0.213 Å^{-1}, and a reciprocal space cutoff of 0.32 Å^{-1}. Also the van der Waals interactions were calculated with a smooth cutoff of 15 Å. For DMOF-1, the van der Waals interactions together with electrostatic interactions are evaluated using the LAMMPS code as a library[31], with van der Waals interactions truncated at 10 Å which is compensated by long-range tail corrections, as in the original work. During the simulations, the temperature was controlled via a single Nosé–Hover chain consisting of three beads and with a relaxation time of 100 fs[32,33,34,35]. Both the particles and the barostat were coupled to the same thermostat. The Martyna–Tobias–Tuckerman–Klein (MTTK) barostat with a relaxation time of 1000 fs was employed to control the pressure[36,37]. This combination of relaxation times ensures that the complete phase space is sampled efficiently, as shown previously[27].

For the simulations of Al-fumarate, MIL-53(Al) and MIL-53(Ga) carried out in Figure 4a, a 200 ps equilibration time was discarded, and averages were taken over a 900 ps equilibration run. For the simulations of MIL-53(Al)-F carried out in Figure 4(a,b), a 100 ps equilibration run was followed by a 1150 ps production run. The simulations for DUT-49(Cu) in Figure 4a were performed using an equilibration time of 50 ps, followed by a 950 ps production run. The simulations for Co(bdp) in Figure 4c were performed using a 100 ps equilibration run followed by a 1400 ps production run. For the simulations of DMOF-1 in Figure 4d, as well as in Supplementary Note 7 of this supporting information, a 200 ps equilibration time was discarded and averages were taken over a 800 ps production run. Finally, for the simulations of xenon in MIL-53(Al) in Supplementary Note 4, of this supporting information, a 100 ps equilibration time was discarded and averages were taken over a 700 ps production run.

Supplementary Note 9 - Entropy contribution to the free energy

In this section, we investigte how the entropy varies in terms of the volume by computing the internal energy in each of the (NV$\sigma_a = 0$T) simulations and computing the entropy contribution by means of the formula $S = \frac{E-F}{T}$. We perform such analysis for various temperatures for MIL-53(Al)-F (simulations of Figure 4b of the manuscript) as well as for various amounts of xenon adsorbed in MIL-53(Al) (simulations of Supplementary Note 4 of this supporting information). Such analysis should allow to give more insight into the larger contribution of entropy to the large pore phase, which is thought to be governing the phase transformation.

MIL-53(Al) with xenon

A decomposition of the free energy into the internal energy and entropy is performed for various xenon loadings, the results are shown in Supplementary Figure 10. The internal energy clearly depends on the number of xenon atoms, which is related to the contributions from the xenon-host and xenon-xenon interactions. Also the entropy is heavily dependent on the number of particles, as expected since higher loadings mean less free space and hence lower entropy.

MIL-53(Al)-F at various temperature

Supplementary Figure 11 shows the decomposition of the free energy of the MIL-53(Al)-F into the internal energy and the entropy contribution for the force-field based MD simulations at various temperatures. Note that the reference levels for the free energy, internal energy and entropy are arbitrarily chosen to enable a visualization of all curves on the same plot. Hence only energy/entropy differences between different volumes are meaningful. The internal energy is almost independent of the temperature (around 3 kJ/mol variation on the lp-cp stability). Interestingly, the entropy variation in terms of the volume is nearly independent on the temperature. However, as expected, the entropy for the large pore phase is substantially higher than in the contracted pore phase ($S(V_{lp}) - S(V_{cp}) \approx 33\ \frac{\mathrm{J}}{\mathrm{mol \cdot K}}$ for a large pore volume $\mathrm{V_{lp}}$ and contracted pore volume $\mathrm{V_{cp}}$ taken as the 300 K equilibria). At higher temperatures, the large pore phase is indeed systematically more stable compared to the contracted pore phase, since the entropy contribution $-T\left(S(V_{lp}) - S(V_{cp})\right)$ to the free energy becomes more dominant due to its linear dependency on the temperature and yields a systematically larger contribution to the overall free energy.

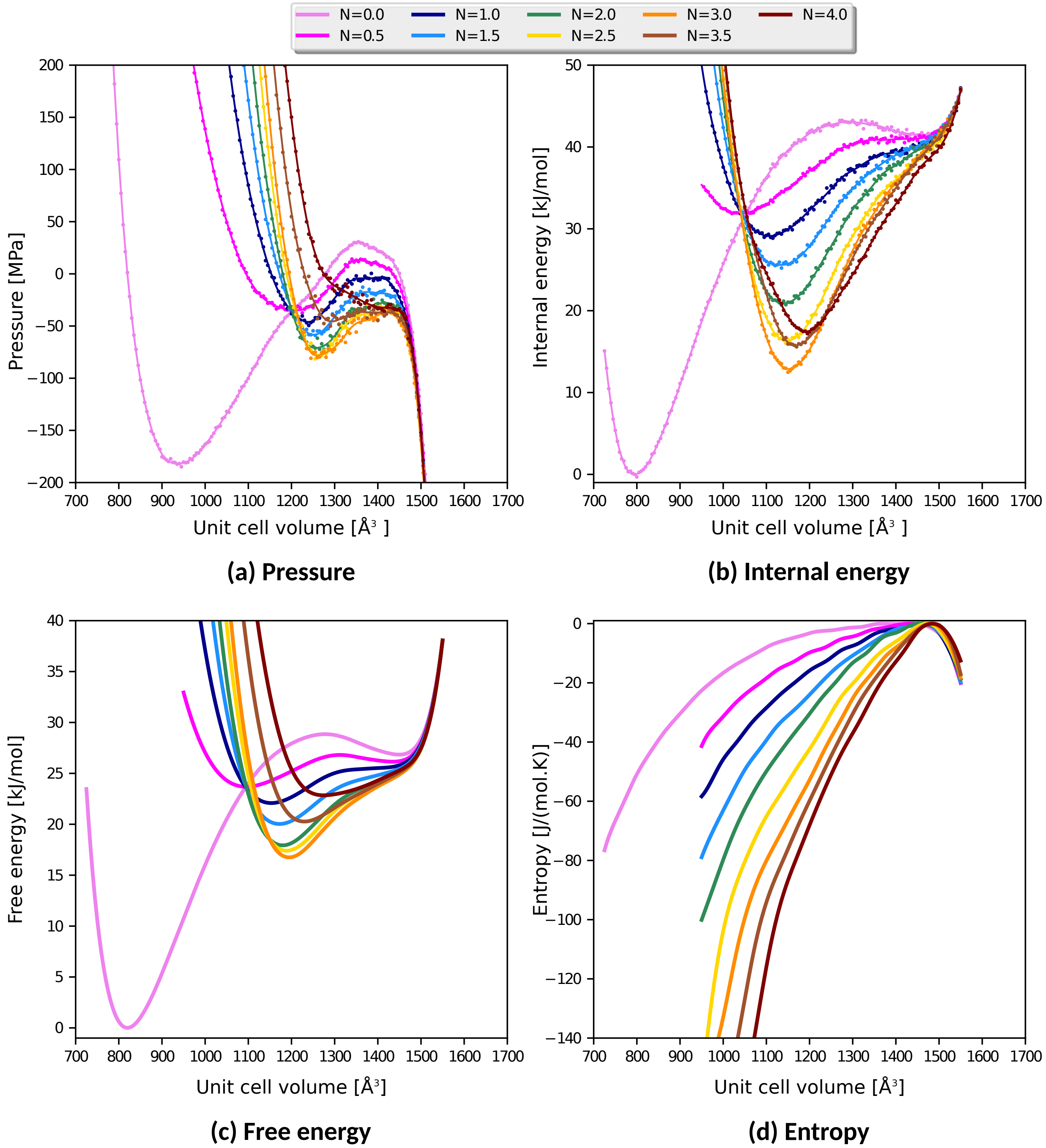

Supplementary Figure 10: MD profiles for various amounts of xenon adsorbed in MIL-53(Al). The upper row shows pressure (upper left) and internal energy (upper right) as function of volume, which are computed directly from the MD simulations for various numbers of xenon atoms adsorbed per unit cell. The lower row shows the free energy (lower left) and entropy (lower right), which are derived by means of thermodynamic integration and the formula $S = \frac{E-F}{T}$ respectively. The curves for the free energy, internal energy and entropy have been shifted, hence, only differences within the same curve are meaningful.

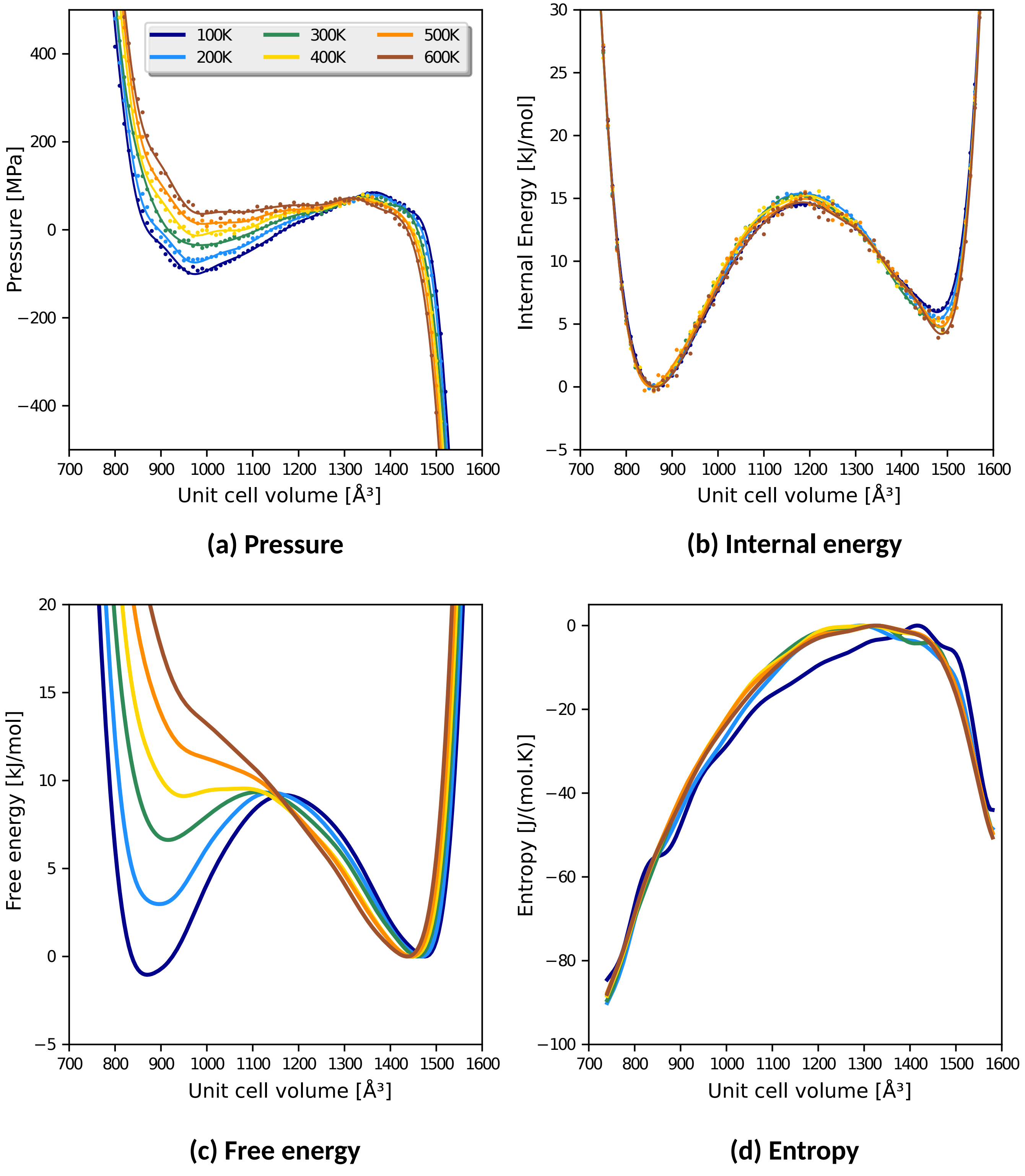

(a) Pressure

(b) Internal energy

(c) Free energy

(d) Entropy

Supplementary Figure 11: (a) Pressure as well as (b) internal energy as function of volume from force-field based MD simulations in the (NV$\sigma_a = 0$T) ensemble. From these profiles, (c) the free energy and (d) entropy profiles are derived using thermodynamic integration and the relation $S = \frac{E-F}{T}$ respectively.

Supplementary Note 10 - Quasi-harmonic approximation

In this section, we calculated the vibrational entropy and free energy via lattice dynamics calculations, also refered to as the quasi-harmonic approximation, and compare the results with the computations from the manuscript based on molecular dynamics simulations. More information on the procedure may be found in the reviews of Butler et al. and Tkatchenko et al.[38,39] This approach is critically dependent on the accurate calculation of the Hessian matrix, which is called the dynamical matrix for periodic systems. It contains the second order derivatives with respect to the geometry of the system. As a result one obtains a series of phonon modes and phonon eigenvectors. To properly include all non-negligible lattice modes a supercell approach might be necessary, as it is important not to introduce artifacts between periodic images.

To obtain thermal corrections at various volume points we applied the quasi-harmonic approximation, where a series of structures are optimized for a number of volume points. The phonons are still harmonic in this case but thermal corrections which are dependent on the volume are taken into account as the phonons become volume dependent. We applied this procedure both for MIL-53(Al) as well as MIL-53(Al) containing 4 xenon atoms, using force fields. In case of force field calculations, the Hessian and forces can be calculated in an analytical way and is thus less prone to numerical uncertainty. We used a $1 \times 2 \times 1$ unit cell which is necessary to describe all potential energy terms correctly in the force field expression. Calculations on a $2 \times 4 \times 2$ unit cell provide very similar results, from which we conclude that the $1 \times 2 \times 1$ supercell is sufficiently large. We choose a grid spacing of 25 Å^3. The results are shown in Supplementary Figure 12. For MIL-53(Al) we find that the quasi-harmonic

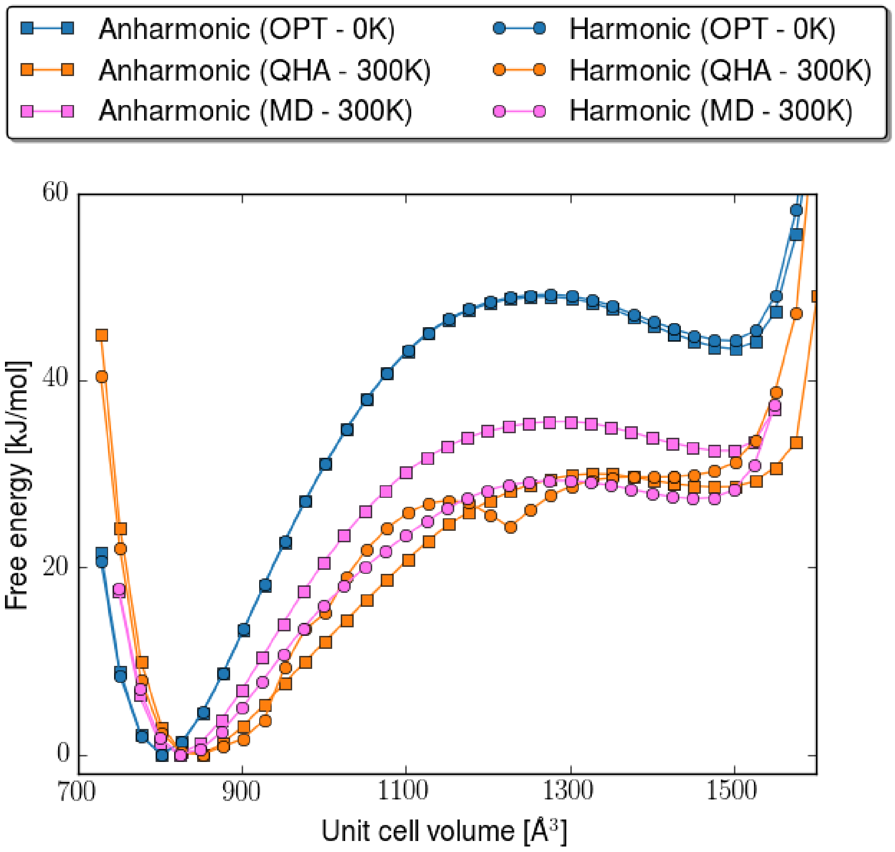

Supplementary Figure 12: Energy profiles for MIL-53(Al) computed with both a harmonic and an anharmonic force field. The blue curves (OPT) represent energies of optimized structures without thermal corrections. The orange (QHA) and pink (MD) curves show the free energy computed within the quasi-harmonic approximation and with thermodynamic integration respectively.

approximation yields fairly good agreement with the MD based results, at least if one uses a force field in which the bonds and bends are described using harmonic terms (which is the case in the submitted version of the paper). To investigate in how far this influences the obtained profiles, we also constructed similar profiles including anharmonic contributions, by adding anharmonic corrections from the MM3 force field to the bond

and bend terms.33 It is clear that the quasi-harmonic profile is in this case in worse agreement with the profile obtained from MD simulations, as only in the latter approach it is possible to correctly sample anharmonicities of the potential energy surface.

For the xenon@MIL-53(Al) system the quasi-harmonic oscillator approximation fails to describe the free energy profile accurately. This can be ascribed to two effects. First of all, it is much more difficult to get smooth normal mode frequencies as function of volume because the xenon guest molecules are only very weakly bound to the framework. This can be observed by the discontinuities in the QHA data points. Second, due to the weak bonding of xenon molecules to the framework, the xenon molecules are able to sample the entire pore of the framework, giving rise to large entropy contributions. However, this high translational degree of freedom is not captured by QHA in which the xenon atoms are restricted to a small region around their equilibrium positions.

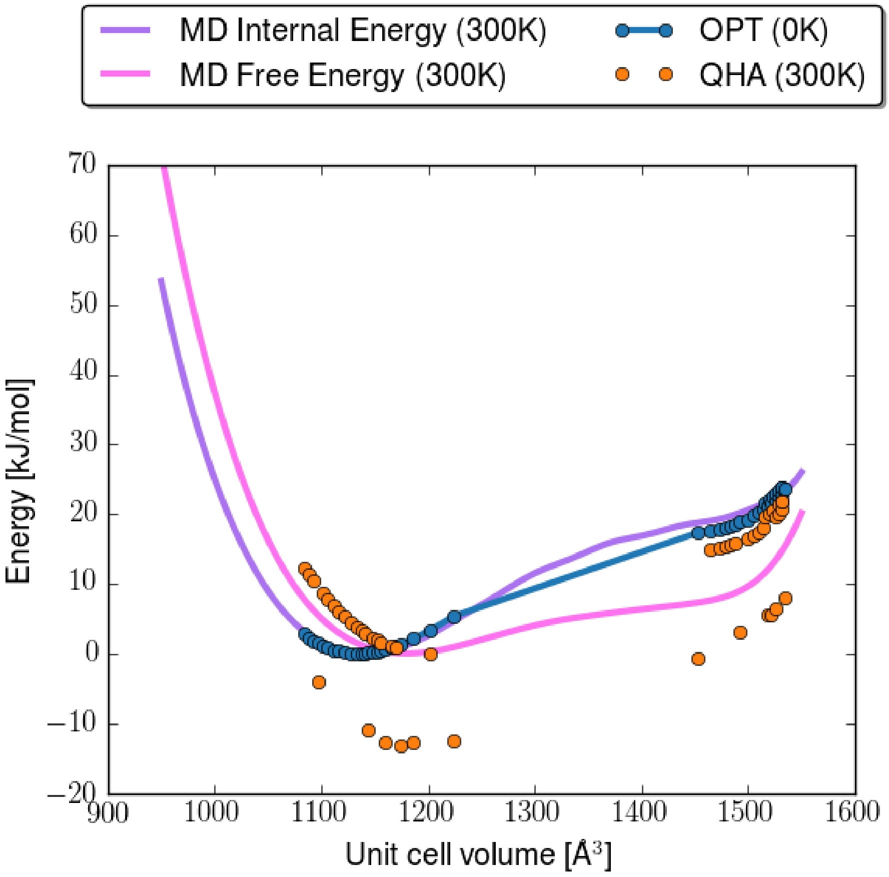

Supplementary Figure 13: Energy profiles for MIL-53(Al) containing 4 xenon atoms computed with the force field from the manuscript. The red dots (FF at 0K) represent internal energies of optimized structures without thermal corrections, the orange curve represents the internal energy at 300K calculated from the MD simulations. The blue dots (QHA-FF at 300K) and green curve (MD Free energy FF at 300K) shows the free energy computed within the quasi-harmonic approximation and with thermodynamic integration respectively.

Supplementary Note 11 - Sensitivity towards simulation time

To study the convergence of our simulations as a function of the simulation time, we have carried out longer simulations for MIL-53(Al)-F at 100 K, 300 K and 600 K, extending the simulation up to 5 ns compared to the previously reported 1.15 ns simulations while keeping the equilibration time of 100 ps constant. We have chosen this material for this in-depth study such that we can also investigate the effect of temperature on the convergence of the simulations. For these three temperatures, the root-mean-square deviation (RMSD) was calculated for three key properties, using the results from the 5 ns simulation as the reference: (i) the pressure profile before fitting, i.e. containing the raw data obtained from the MD simulation, (ii) the pressure profile after fitting, and (iii) the free energy profile obtained by thermodynamic integration of the pressure profile. The resulting RMSDs are reported in Supplementary Table 3 (100 K), 4 (300 K), and 5 (600 K). Across all

Supplementary Table 3: Root-mean-square deviation (RMSD) of the pressure profile (both before and after fitting) and free energy profile for MIL-53(Al)-F at 100 K, using the 5 ns simulations as a reference. Values reported in the main text were obtained for a simulation run of 1.15 ns.

	**0.5 ns**	**1 ns**	**2 ns**	**3 ns**	**4 ns**
**Pressure RMSD before fit [MPa]**	5.58	3.49	1.55	1.13	0.96
**Pressure RMSD after fit [MPa]**	3.46	2.06	0.63	0.36	0.33
**Free energy RMSD [kJ/mol]**	0.23	0.10	0.02	0.02	0.02

Supplementary Table 4: Root-mean-square deviation (RMSD) of the pressure profile (both before and after fitting) and free energy profile for MIL-53(Al)-F at 300 K, using the 5 ns simulations as a reference. Values reported in the main text were obtained for a simulation run of 1.15 ns.

	**0.5 ns**	**1 ns**	**2 ns**	**3 ns**	**4 ns**
**Pressure RMSD before fit [MPa]**	5.86	4.06	2.64	1.79	1.10
**Pressure RMSD after fit [MPa]**	1.88	1.03	1.16	0.99	0.51
**Free energy RMSD [kJ/mol]**	0.12	0.05	0.02	0.02	0.05

Supplementary Table 5: Root-mean-square deviation (RMSD) of the pressure profile (both before and after fitting) and free energy profile for MIL-53(Al)-F at 600 K, using the 5 ns simulations as a reference. Values reported in the main text were obtained for a simulation run of 1.15 ns.

	**0.5 ns**	**1 ns**	**2 ns**	**3 ns**	**4 ns**
**Pressure RMSD before fit [MPa]**	8.30	5.23	2.94	2.03	1.25
**Pressure RMSD after fit [MPa]**	2.39	1.28	0.77	0.64	0.45
**Free energy RMSD [kJ/mol]**	0.10	0.07	0.06	0.06	0.04

temperatures, we observe an RMSD of 3-6 MPa on the pressure before fitting when comparing the 1 ns simulation (indicated in gray, close to the 1.15 ns simulations reported in the manuscript) with the longer 5 ns simulation (assumed to be converged), which slightly increases with increasing temperature (3.5 MPa at 100 K versus 5.2 MPa at 600 K). This may be attributed to the size of the available phase space, which increases at higher temperatures, whereas the slower exploration of the phase space at 100 K, due to the lower velocities, do not lead to worse sampling. By fitting the pressure, the RMSD drops to about 1-2 MPa for all temperatures, well within the accuracy one can expect to obtain with this method. This RMSD in the pressure results in an RMSD in the free energy of about 0.1 kJ/mol when comparing the 1 ns simulation with the 5 ns simulation.

To get more insight in which volume regions of the pressure and free energy profiles are most prone to these small imprecisions, we report in Supplementary Figure 14 the pressure profile before fitting (pane a) and the free energy profile obtained by thermodynamic integration (pane c) as a function of the unit cell volume for the six different simulation times at 300 K. Since the different profiles are virtually indistinguishable on this scale, panes (b) and (d) report the difference in pressure, respectively free energy, with respect to the 5 ns simulation. We observe that the deviations decrease with increasing simulation time, and that these deviations are the largest at the smallest volumes. Finally, in Supplementary Table 6 (100 K), 7 (300 K), and 8 (600 K), the volumes of

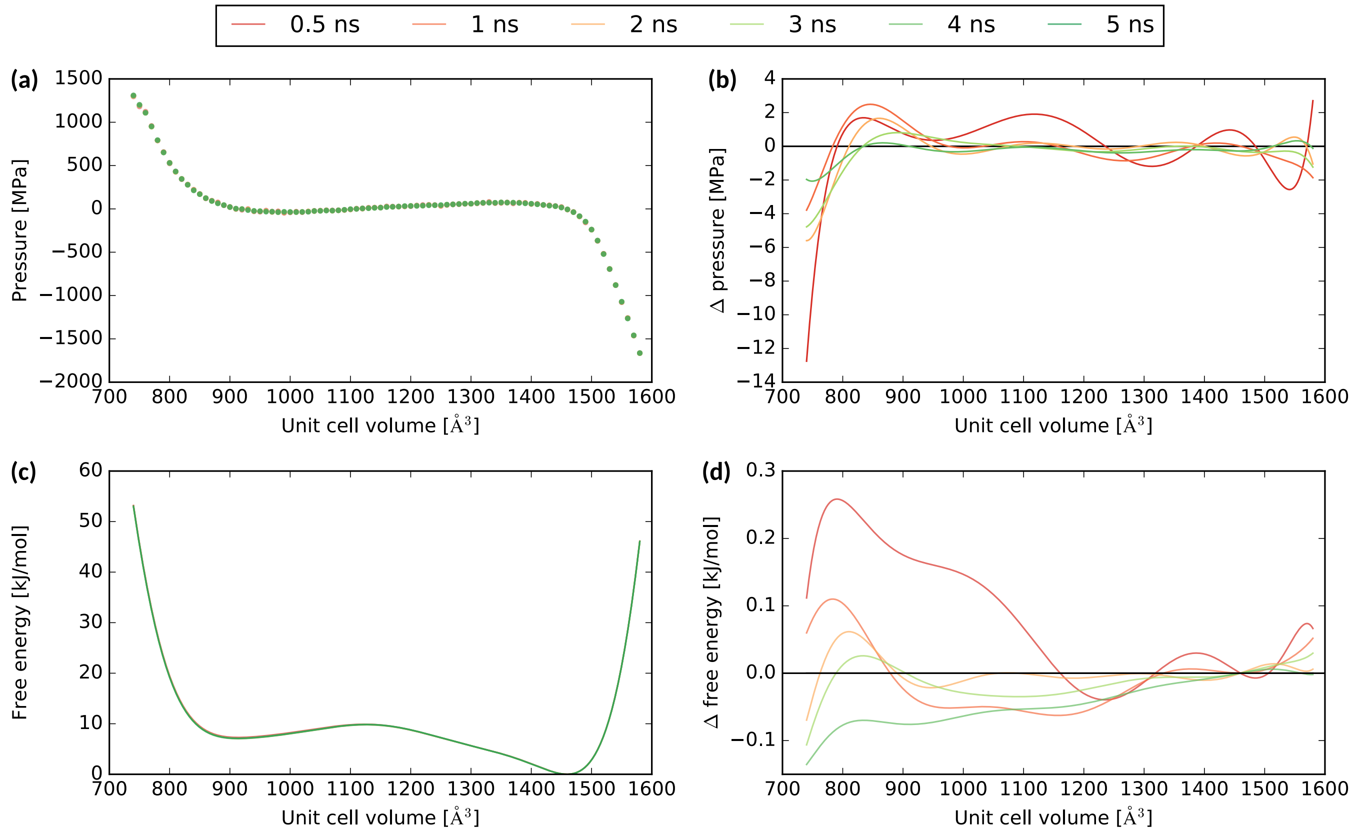

Supplementary Figure 14: Obtained pressure and free energy profiles for MIL-53(Al)-F at 300 K with different simulation times: (a) Pressure profile before fitting, (b) Difference in pressure profiles using the 5 ns simulation as reference, (c) Free energy profile, (d) Difference in free energy profiles using the 5 ns simulations as reference.

the metastable CP and LP states as well as the LP-to-CP and CP-to-LP transition pressures extracted from the pressure profiles with different simulation times are reported. We observe that the deviations due to limited simulation time do only affect these properties to a minor extent. In conclusion, the earlier reported 1.15 ns simulations have converged sufficiently to yield accurate results within a few MPa and a few tenths kJ/mol for the pressure and free energy profiles, respectively, yielding volumes for the metastable states which are accurate within a few Å^3.

Supplementary Table 6: Volumes of the metastable CP and LP states and the two transition pressures for MIL-53(Al)-F at 100 K as extracted from MD simulations with different simulation times (0.5 - 5 ns). Values reported in the main text were obtained for a simulation run of 1.15 ns.

	0.5 ns	1 ns	2 ns	3 ns	4 ns	5 ns
CP volume [Å³]	872	870	871	870	870	870
LP volume [Å³]	1471	1471	1471	1471	1471	1471
LP-to-CP transition pressure [MPa]	87.4	87.1	87.1	87.1	87.0	87.1
CP-to-LP transition pressure [MPa]	-81.6	-81.2	-81.4	-80.7	-80.7	-80.9

Supplementary Table 7: Volumes of the metastable CP and LP states and the two transition pressures for MIL-53(Al)-F at 300 K as extracted from MD simulations with different simulation times (0.5 - 5 ns). Values reported in the main text were obtained for a simulation run of 1.15 ns.

	0.5 ns	1 ns	2 ns	3 ns	4 ns	5 ns
CP volume [Å³]	914	915	914	914	913	913
LP volume [Å³]	1460	1459	1459	1459	1459	1459
LP-to-CP transition pressure [MPa]	77.8	77.5	77.2	77.2	77.1	77.3
CP-to-LP transition pressure [MPa]	-30.0	-31.2	-31.4	-31.0	-31.4	-31.2

Supplementary Table 8: Volumes of the metastable CP and LP states and the two transition pressures for MIL-53(Al)-F at 600 K as extracted from MD simulations with different simulation times (0.5 - 5 ns). Values reported in the main text were obtained for a simulation run of 1.15 ns.

	0.5 ns	1 ns	2 ns	3 ns	4 ns	5 ns
CP volume [Å³]	1020	1020	1020	1020	1020	1020
LP volume [Å³]	1441	1441	1441	1441	1441	1441
LP-to-CP transition pressure [MPa]	70.3	69.4	68.6	68.3	67.9	67.8
CP-to-LP transition pressure [MPa]	27.4	29.3	29.4	29.1	29.1	28.8

Supplementary Note 12 - Description of available data upon request

Following files are available upon request from the authors:

- force_fields.zip: for each material for which a QuickFF force field was constructed in this work (see Supplementary Note 6), a pars.txt file containing the force field parameters in Yaff format, as well as an initial structure in Yaff CHK and XYZ format is included.
- simresults.zip: contains the pressure versus volume data points resulting from the $(N, V, \sigma_a = 0, T)$ simulations, as well as the energy versus volume data points for xenon@MIL-53(Al) and MIL-53(Al)-F (see Supplementary Note 9).

Supplementary References

[1] Rogge, S. M. J. *et al.* Thermodynamic insight in the high-pressure behavior of uio-66: Effect of linker defects and linker expansion. *Chem. Mater.* **28**, 5721–5732 (2016).

[2] Boutin, A. *et al.* Breathing Transitions in MIL-53(Al) Metal-Organic Framework Upon Xenon Adsorption. *Angewandte Chemie International Edition* **48**, 8314–8317 (2009).

[3] Vanduyfhuys, L., Ghysels, A., Rogge, S., Demuynck, R. & Van Speybroeck, V. Semi-analytical mean-field model for predicting breathing in metal-organic frameworks. *Mol. Simulat.* **41**, 1311–1328 (2015).

[4] Krause, S. *et al.* A pressure-amplifying framework material with negative gas adsorption transitions. *Nature* **532**, 348–52 (2016).

[5] Evans, J. D., Bocquet, L. & Coudert, F.-X. Origins of negative gas adsorption. *Chem* **1**, 873–886 (2016).

[6] Vanduyfhuys, L. *et al.* Quickff: A program for a quick and easy derivation of force fields for metal-organic frameworks from ab initio input. *J. Comput. Chem.* **36**, 1015–1027 (2015).

[7] Allinger, N., Yuh, Y. & Lii, J. Molecular mechanics. the mm3 force field for hydrocarbon 3. 1. *J. Am. Chem. Soc.* **111**, 8551–8566 (1989).

[8] Allinger, N. L., Zhou, X. & Bergsma, J. Molecular mechanics parameters. *J. Mol. Struc-Theochem* **312**, 69–83 (1994).

[9] Grosch, J. & Paesani, F. Molecular-level characterization of the breathing behavior of the jungle-gym-type DMOF-1 metal-organic framework. *J. Am. Chem. Soc.* **134**, 4207–4215 (2012).

[10] Addicoat, M. A., Vankova, N., Akter, I. F. & Heine, T. Extension of the universal force field to metal–organic frameworks. *J. Chem. Theory Comput.* **10**, 880–891 (2014).

[11] Boyd, P. G., Moosavi, S. M., Witman, M. & Smit, B. Force-field prediction of materials properties in metal-organic frameworks. *J Phys Chem Lett* **8**, 357–363 (2017).

[12] Kresse, G. & Furthmüller, J. Efficiency of ab-initio total energy calculations for metals and semiconductors using a plane-wave basis set. *Comp. Mat. Sci.* **6**, 15–50 (1996).

[13] Perdew, J., Burke, K. & Ernzerhof, M. Generalized gradient approximation made simple. *Phys. Rev. Lett.* **77**, 3865–3868 (1996).

[14] Perdew, J., Burke, K. & Ernzerhof, M. Generalized gradient approximation made simple. *Phys. Rev. Lett.* **78**, 1396–1396 (1997).

[15] Grimme, S., Antony, J., Ehrlich, S. & Krieg, H. A consistent and accurate ab initio parametrization of density functional dispersion correction (DFT-D) for the 94 elements h-pu. *J. Chem. Phys.* **132**, 154104 (2010).

[16] Grimme, S., Ehrlich, S. & Goerigk, L. Effect of the damping function in dispersion corrected density functional theory. *J. Comput. Chem.* **132**, 1456–1465 (2011).

[17] Vinet, P., Ferrante, J., Rose, J. & Smith, J. Compressibility of solids. *J. Geophys. Res.* **92**, 9319–9325 (1987).

[18] Vanpoucke, D., Lejaeghere, K., Van Speybroeck, V., Waroquier, M. & Ghysels, A. Mechanical properties from periodic plane wave quantum mechanical codes: The challenge of the flexible nanoporous MIL-47(V) framework. *J. Phys. Chem. C* **119**, 23752–23766 (2015).

[19] Verstraelen, T. *et al.* Minimal basis iterative stockholder: Atoms in molecules for force-field development. *J. Chem. Theory Comput.* **12**, 3894–3912 (2016).

[20] Mortensen, J., Hansen, L. & Jacobsen, K. Real-space grid implementation of the projector augmented wave method. *Phys. Rev. B* **71**, 035109–035119 (2005).

[21] Enkovaara, J. *et al.* Electronic structure calculations with GPAW: a real-space implementation of the projector augmented-wave method. *J. Phys.-Condens. Mat.* **22**, 253202–253225 (2010).

[22] Bahn, S. & Jacobsen, K. An object-oriented scripting interface to a legacy electronic structure code. *Comput. Sci. Eng.* **4**, 56–66 (2002).

[23] Chen, J. & Martínez, T. J. Qtpie: Charge transfer with polarization current equalization. a fluctuating charge model with correct asymptotics. *Chem. Phys. Lett.* **438**, 315–320 (2007).

[24] Wang, J., Wolf, R., Caldwell, J., Kollman, P. & Case, D. Development and testing of a general amber force field. *J. Comput. Chem.* **25**, 1157–1174 (2004).

[25] Truhlar, Y. Z. . D. The m06 suite of density functionals for main group thermochemistry, thermochemical kinetics, noncovalent interactions, excited states, and transition elements: two new functionals and systematic testing of four m06-class functionals and 12 other functionals. *Theor. Chem. Acc.* 215–241 (2008).

[26] Breneman, C. & Wiberg, K. Determining atom-centered monopoles from molecular electrostatic potentials - the need for high sampling density in formamide conformational-analysis. *J. Comput. Chem.* **11**, 361–373 (1990).

[27] Rogge, S. M. J. *et al.* A comparison of barostats for the mechanical characterization of metalorganic frameworks. *J. Chem. Theory Comput.* **11**, 5583–5597 (2015).

[28] Verstraelen, T., Vanduyfhuys, L., Vandenbrande, S. & Rogge, S. M. J. *Yaff, Yet Another Force Field*, http://molmod.ugent.be/software/.

[29] Smith, W. & Forester, T. Dl_poly_2.0: a general-purpose parallel molecular dynamics simulation package. *J. Mol. Graph.* **14**, 136–141 (1996).

[30] Bureekaew, S. & Schmid, R. Hypothetical 3d-periodic covalent organic frameworks: exploring the possibilities by a first principles derived force field. *CrystEngComm* **15**, 1551 (2013).

[31] Plimpton, S. Fast parallel algorithms for short-range molecular dynamics. *J. Comput. Phys.* **117**, 1–19 (1995).

[32] Nosé, S. A molecular dynamics method for simulations in the canonical ensemble. *Mol. Phys.* **52**, 255–268 (1984).

[33] Nosé, S. A unified formulation of the constant temperature molecular dynamics methods. *J. Chem. Phys.* **81**, 511–519 (1984).

[34] Hoover, W. G. Canonical dynamics: Equilibrium phase-space distributions. *Phys. Rev. A* **31**, 1695–1697 (1985).

[35] Martyna, G. J., Klein, M. L. & Tuckerman, M. Nosé-hoover chains: The canonical ensemble via continuous dynamics. *J. Chem. Phys.* **97**, 2635–2643 (1992).

[36] Martyna, G. J., Tobias, D. J. & Klein, M. L. Constant pressure molecular dynamics algorithms. *J. Chem. Phys.* **101**, 4177–4189 (1994).

[37] Martyna, G. J., Tuckerman, M. E., Tobias, D. J. & Klein, M. L. Explicit reversible integrators for extended systems dynamics. *Mol. Phys.* **87**, 1117–1157 (1996).

[38] Butler, K. T., Walsh, A., Cheetham, A. K. & Kieslich, G. Organised chaos: entropy in hybrid inorganic-organic systems and other materials. *Chem. Sci.* **7**, 6316–6324 (2016).

[39] Hoja, J., Reilly, A. M. & Tkatchenko, A. First-principles modeling of molecular crystals: structures and stabilities, temperature and pressure. *Wiley Interdiscip. Rev.-Comput. Mol. Sci.* **7**, 26 (2017).
